# Strategic Switching from Conventional Urea to Nano-Urea for Sustaining the Rice–Wheat Cropping System

**DOI:** 10.3390/plants13243523

**Published:** 2024-12-17

**Authors:** Ashwani Kumar, Parvender Sheoran, Sunita Devi, Naresh Kumar, Kapil Malik, Manu Rani, Arvind Kumar, Pooja Dhansu, Shruti Kaushik, Ajay Kumar Bhardwaj, Anita Mann, Rajender Kumar Yadav

**Affiliations:** 1 ICAR—Central Soil Salinity Research Institute, Karnal 132001, India; khatkersunita@gmail.com (S.D.); drnareshbiochem@eternaluniversity.edu.in (N.K.); kapilmalik017@hau.ac.in (K.M.); manusagwal@gmail.com (M.R.); arvind.kumar2@icar.gov.in (A.K.); shrutikaushik22@gmail.com (S.K.); ak.bhardwaj@icar.gov.in (A.K.B.); anita.mann@icar.gov.in (A.M.); rk.yadav@icar.gov.in (R.K.Y.); 2 ICAR—Agricultural Technology Application Research Institute, Ludhiana 141004, India; 3 Department of Chemistry and Biochemistry, Eternal University, Baru Sahib 171101, India; 4 ICAR—Sugarcane Breeding Institute, Regional Centre, Karnal 132001, India; pooja@icar.gov.in

**Keywords:** nano-nitrogen, physiological traits, yield, N-metabolizing enzymes, available soil N

## Abstract

In the face of declining crop yields, inefficient fertilizer usage, nutrient depletion, and limited water availability, the efficiency of conventional NPK fertilizers is a critical issue in India. The hypothesis of this study posits that nano-nitrogen could enhance growth and photosynthetic efficiency in crop plants compared to conventional fertilizers. For this, a randomized block design (RBD) field experiment was conducted with six treatments: no nitrogen (T1), 100% N through urea (T2), and varying levels of N replacement with nano-nitrogen (33%: T3; 50%: T4; 66%: T5; and 100%: T6). Morphological and physiological traits and yield attributes were measured at physiological maturity, and yield attributes were measured at harvest. Results showed that 33% nitrogen replacement with nano-nitrogen (T3) outperformed conventional urea (T2) in physiological traits and achieved higher grain yields (3789 kg/ha for rice and 4206 kg/ha for wheat) compared to T2 (3737 kg/ha for rice and 4183 kg/ha for wheat with 100% urea). Although T4 and T5 showed statistically similar yields, they were lower than T2 and T3 for rice, while 50%, 66%, and 100% replacements reduced wheat yield by 2.49%, 8.39%, and 41.26%, respectively, compared to T2. Key enzymes of N metabolism decreased with higher nano-nitrogen substitution. Maximum nitrogen availability was observed in T2 and T3. This study concludes that nano-nitrogen is an effective strategy to enhance growth, balancing productivity and environmental sustainability.

## 1. Introduction

Agriculture is a major source of income in emerging nations’ rural areas, contributing towards the economic development of a nation. To boost agricultural productivity, applying fertilizers in the soil is a standard technique for increasing crop yield to meet the ever-increasing demands of the growing world population with environmental security. Additionally, increasing the efficiency of applied fertilizers and reducing the contamination caused by over fertilization are critical goals for sustainable agricultural development. Despite advances in our knowledge of how plants assimilate nutrients effectively, there are still no fertilizers that successfully supply the ideal plant nutrients or are used to their full potential. It is estimated that less than half of the applied fertilizers are actually used by the crops, with the remainder being lost to the environment [1,2].

Nitrogen, a major constituent of NPK, is a structural component of many enzymes, proteins, and chlorophyll which is essential for vegetative development of crops. From just 0.21 Mt in 1960–1961 to 17.63 Mt in 2018–2019, fertilizer N consumption in India’s food-grain-driven agriculture has increased dramatically [3]. The optimal NPK ratio of 4:2:1 is ideal for crop productivity but farmers are using NPK fertilizer in a ratio of 10:2.7:1. This imbalance may be due to over estimations of nitrogen benefits in crop productivity, as well as misconceptions about their interactions, causing farmers to abuse N fertilizers [4]. Hence, improving nitrogen use efficiency in agricultural farming systems would be advantageous for the world’s sustainable food supply and environmental security [5,6].

Recent advances in nanotechnology with controlled release techniques and targeted delivery of agrochemicals (fertilizers, herbicides, insecticides, pesticides) for plant nutrition and disease control offer promising opportunities for efficient utilization of fertilizers/chemicals. Among these advances, there is a search for new coated fertilizers as urea granules coated with various materials that will enhance nitrogen stay in soil and enhance its efficiency and recovery or by the use of nano-fertilizers. In this context, the strategic switching from conventional urea to nano-urea represents a promising solution for sustaining the rice–wheat cropping system. Nano-urea, with its higher nutrient use efficiency, can potentially reduce the environmental impact of fertilizer use while maintaining or even enhancing crop productivity. This innovation not only addresses the inefficiencies of traditional fertilizers but also aligns with the goals of sustainable agricultural practices by reducing nitrogen losses to the environment and promoting more precise nutrient management.

Recently, in 2019, IFFCO released nano-urea (commercialized by the Govt. of India) containing 85–99.98% urea, 0.01% hydroquinone, and 0.01% calcium cyanamide. Nano-urea has the potential to reduce the usage of conventional chemical fertilizers besides raising crop output as well as efforts to cut usage of chemical fertilizers and boost farmers’ income [1]. Since the application of fertilizer N is relatively higher than the recommended dose in salt-affected soils, the application of nano-urea and other sources may become a better and more energy-efficient dose of fertilizer. Also, the foliar application of plant nutrients has potential advantages over soil application by increasing the use efficiency of fertilizers which, somehow, also helps in alleviating the physiological stress to plants in stressful conditions. Keeping these facts in view, the present experiment was planned to investigate and explore the integration and substitution effects of nano-nitrogen on the rice–wheat cropping system. The research hypothesis posited that nano-nitrogen would enhance growth and productivity compared to conventional fertilizers. The broader goal of the study was to demonstrate alternative practices for sustainable and precise agriculture that could improve economic efficiency and enhance farmers’ income.

## 2. Results

### 2.1. Morphological Traits

Substitution of urea through nano-N resulted in non-significant differences in plant height after up to 50% replacement of urea in rice and 66% replacement of urea in wheat (Table 1). Treatment T3 receiving ⅔ N through urea and ⅓ N through nano-N resulted in higher plant height both in rice and wheat in comparison to T2. Beyond 33% substitution, rice and wheat plants showed a decline in plant height. CI (chlorophyll index measures greenness) and NBI (nitrogen balance index measures the ratio between chlorophyll to flavanols for assessing the nitrogen) were measured in randomly selected leaves of rice and wheat crops. CI values were found maximally higher in T2 (35.99) followed by T3 (33.96), T4 (33.7), and T5 (33.34) in rice. Whereas in the case of wheat, higher CI was noted in T3 (30.25) followed by T2 (28.02). In both rice and wheat crops, CI values were lowest in T1 and T6 (Table 1). A similar trend of values was noticed for NBI which reflects the nitrogen nutritional status of plants, being maximum under T3 and T2 (Table 1). Treatment T1 receiving no-N showed the lowest NBI values in both crops.

### 2.2. Yield Attributes and Yield

The data presented in Table 2 show the changes in yield-attributing traits of rice and wheat crops in response to different N sources. The number of tillers and effective tillers of rice showed significant variability and found maximum tillers/effective tillers in T2 (60.50 and 49.50/mrl) followed by T3 (59.25 and 48.75/mrl). Panicle length was maximum in T3 (10.85 cm) followed by T2 (10.45 cm). Further replacement of urea with nano-N resulted in a decrease in panicle length and found 10.03 cm in T4, 9.53 cm in T5, and 7.85 cm in T6 (Table 2). Panicle weight was also recorded and noted a statistically higher panicle weight of 2.70 g in T3 followed by 2.61 g in T2 and 2.53 g in T4 (Table 2). The number of grains/panicle also showed similar results and found maximum grains/panicle in T3 (104.0) followed by T2 (103.0). Above 33% replacement of N through nano-N showed a significant decline in the number of grains/panicle and noted 94.25 grains in T4, 93.5 grains in T5, and 86.0 grains in T6. Similar results were noted for the number of filled grains/panicles (Table 2). Grain weight/panicle was found to be higher in T3 (2.05 g) which was statistically on par with T2 (2.01 g). Further substitution of urea resulted in a reduction of grain weight/panicle by 7.0%, 17.9%, and 39.8% under T4, T5, and T6 in comparison to T2. The ear-filling ratio in rice was calculated by dividing grain weight/panicle by panicle weight and found higher ear filling under T2 (77%) which was statistically at par with T3 (76%). Thereafter further substitution of urea led to a significant decline in ear-filling ratio (Table 2).

**Figure 1:**
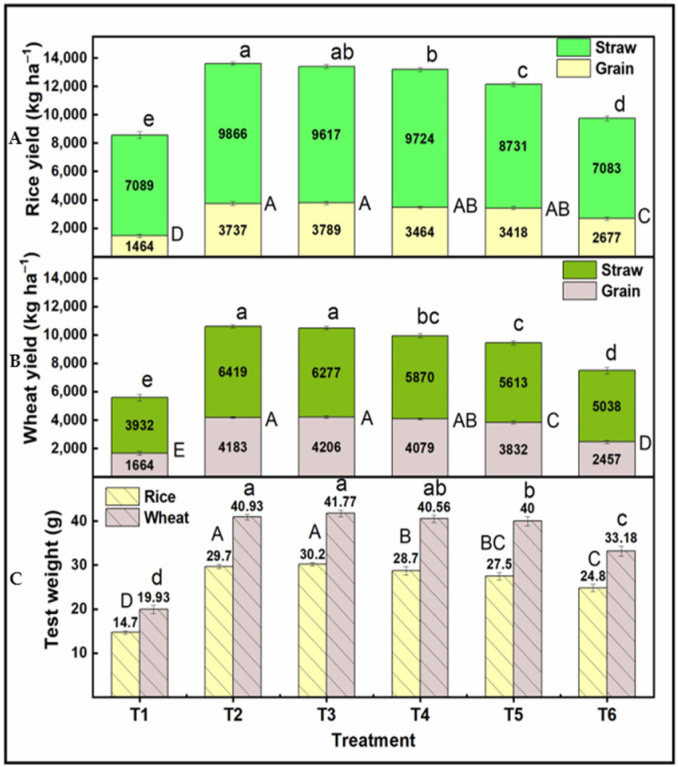
Effect of nitrogen substitution through nano-nitrogen on test weight (g), grain yield (kg/ha), and straw yield (kg/ha) of rice and wheat crop. (**A**)—the lowercase letter represented significant differences between treatments in straw yield of rice crop while uppercase letters represented differences in grain yield of rice crop; (**B**)—the lowercase letter represented significant differences between treatments in straw yield of wheat crop while uppercase letters represented differences in grain yield of wheat crop; (**C**)—the uppercase letter represented significant differences between treatments in Test weight of rice crop while lowercase letters represented differences in Test weight of wheat crop).

Data presented in Figure 1 show 1000-grain weight (test weight) under different N sources and found statistically different results for different N replacements except T2 and T3, which were statistically at par. The 1000 seed weight was 29.7 g under T2, 30.2 g under T3, 28.7 g under T4, 27.5 g under T5, and 24.8 g under T6. Treatment T1 had the lowest test weight of 14.7 g (Figure 1). The effect of nitrogen replacement with nano-N on straw yield and grain yield of PB 1121 (rice) is shown in Figure 1. The maximum straw yield was observed in treatment T2 (9866 kg/ha), followed by T3 (9617 kg/ha). In terms of grain yield, T3 produced the highest yield (3789 kg/ha), closely followed by T2 (3737 kg/ha). Although treatments T4 and T5 yielded statistically similar results, their values were still lower compared to T2 and T3 (Figure 1).

In the case of wheat, a higher number of tillers and effective tillers was recorded under T3, which was statistically at par with T2 and T4 (Table 2). Whereas, the maximum spike length was recorded under the T2 scenario which was reduced by 1.05% under T3, 5.15% under T4, 6.2% under T5, and 19.64% under T6. Similar results were also noted for spike weight (Table 2). Spikelets/spike was also counted, noted that significantly higher spikelets were found under T3 (18.77) followed by T2 (17.90) and T4 (17.53). Nitrogen substitution up to 66% showed non-significant results for the number of grains/spikes but the values were numerically higher under T3 (55.23). Grain weight/spike exhibited significant differences among different N scenarios and noted higher weight under T3 (2.31 g). Treatment T1 and T6 resulted in lower grain weight/spike (Table 2). Substitution of N through nano-N up to 66% showed more than a 75% ear-filling in wheat, being maximum under T3 (81%).

The test weight (1000 grain weight) of KRL 210 (wheat) was marginally on the higher side with the replacement of conventional source through nano-N and found that 33% replacement resulted in maximum test weight of 41.77 g in comparison to RDN through urea (40.93 g) (Figure 1). Further substitution of N through nano-N resulted in a decline in test weight by 0.91% under T4, 2.27% under T5, and 18.9% under the T6 scenario (Figure 1). The higher test weight under nano-N application (33% replacement) was also reflected in yield, i.e., RDN-treated plots produced a grain yield of 4183 kg/ha which increased to 4206 kg/ha under T3 (Figure 1). Further increase in N replacement (50%, 66%, and 100% replacement) yielded 2.49%, 8.39%, and 41.26% reduction compared to recommended N through urea (T2). Straw yield showed similar results as that obtained for rice.

### 2.3. Physiological Traits

**Figure 2:**
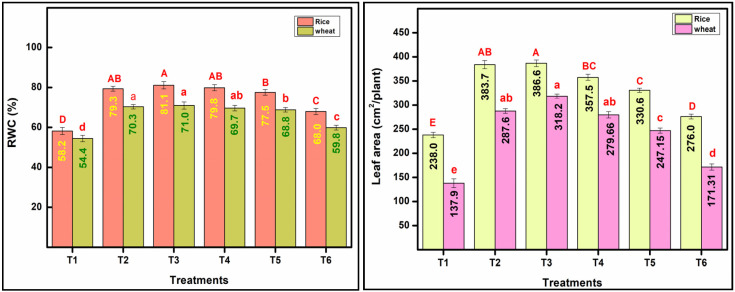
The effect of nitrogen substitution through nano-nitrogen on RWC (%) and Leaf area (cm^2^/plant) of rice and wheat crop (means followed by at least one letter common are not statistically significant (*p* < 0.05) using LSD test). The uppercase letter represented significant differences between treatments in rice crop while lowercase letters represented differences in wheat crop.

Among physiological traits, RWC is an important one that reflects the balance between water supply to the leaf tissue and transpiration rate. Numerically higher RWC was noted under T3 and T4 in rice as compared with T2, but statistically the values were on par. In the case of wheat, statistically equivalent RWC was recorded in T2 (70.39%) and T3 (71.04%). Beyond 33% substitution of urea with nano-N, RWC decreased by 0.94%, 2.13%, and 14.9% under T4, T5, and T6 in comparison to T2 in wheat (Figure 2).

Leaf area (LA) is an important trait that governs photosynthetic efficiency and plant development via potential plant growth, nutrition availability, and yield. The present result signifies that T3 treatment had a higher LA per plant of 386.6 and 318.28 cm^2^ in rice and wheat (Figure 2). In the case of the rice crop, T3 had a higher LA per plant, while in wheat, T3 and T4 had higher plant foliage recording of 318.28 cm^2^/plant and 287.68 cm^2^/plant, a greater leaf area than the recommended N T2 (T2; 279.66 cm^2^/plant). Spectro-photometric measurement of greenness in terms of chlorophyll content also reflected the beneficial effect of 33% substitution of urea with nano-N. Similar to LA, chlorophyll content was also statistically at par in T2 (2.12 mg/g) and T3 (2.15 mg/g) in rice and T2 (2.14 mg/g), T3 (2.17 mg/g), and T4 (2.07 mg/g) in wheat (Table 3). Replacing urea through nano-N showed significant changes in gas exchange traits (Pn, gS, E, and WUEi), but the treatment with one dose replacement (33%) had marginally higher gas exchange traits in comparison to RDN through urea (Table 3). Treatments T4 and T5 replacement showed reductions of 7.78–9.69% in Pn, 18.42–28.95% in gS, and 14.46–18.88% in E in rice crops and 5.78–10.27% in Pn, 12.73–21.82% in gS, and 4.54–7.94% in E in wheat crops as compared with T2 (Table 3). Water use efficiency was found to be non-significant in both rice and wheat. Higher values of WUEi were obtained under T1 and T6 treatments in comparison to other treatments in both crops (Table 3).

### 2.4. Nitrogen-Metabolizing Enzymes

**Figure 3:**
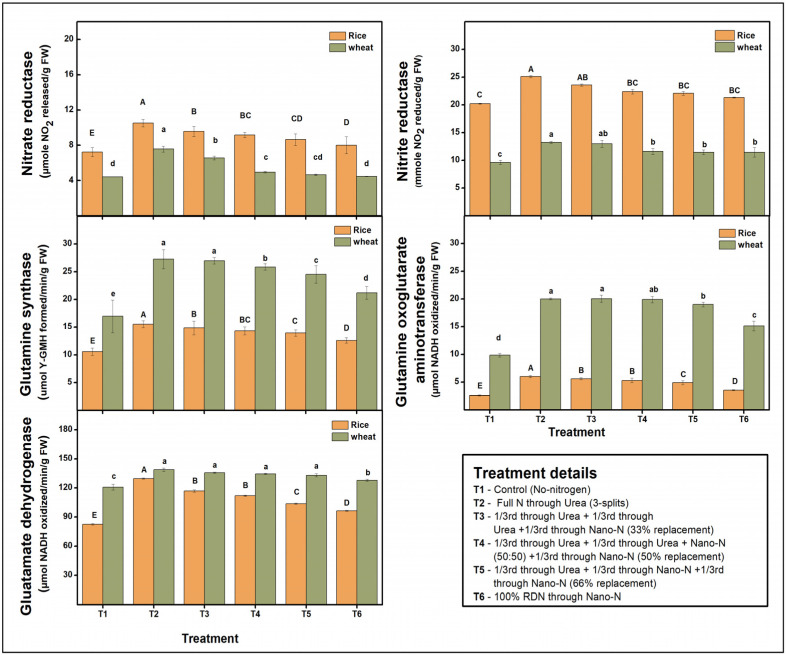
Effect of nitrogen substitution through nano-nitrogen on nitrogen-metabolizing enzymes in rice and wheat crops (means followed by at least one letter common are not statistically significant (*p* < 0.05) using LSD test). The uppercase letter represented significant differences between treatments in rice crop while lowercase letters represented differences in wheat crop.

Nitrate and ammonium nitrogen, the two principal nitrogen sources in plants, perform critical roles in a variety of physiological and metabolic activities. Plant nitrogen assimilation involved several enzymes [nitrate reductase (NR), nitrite reductase (NiR), glutamine synthetase (GS), glutamine synthase/glutamaine-oxoglutarate aminotransferase (GOGAT), and glutamine dehydrogenase (GDH)] and intermediates that contribute towards synthesis and conversion of amino acid through nitrate reduction. The activities of these enzymes were measured after 7 days of complete fertilization application and noted that the activities of studied enzymes were higher under conventional urea practices in both crops (Figure 3). Increased substitution of urea with nano-N resulted in a constant decrease in the activity of these enzymes. In rice under RDN applied through conventional urea, NR activity was 10.51 μmol NO_2_ released/g FW which decreased by 9.04%, 12.65%, 17.7%, and 23.79% under T3, T4, T5, and T6 (Figure 3). NiR activity was 25.12 mmole NO_2_ reduced/g FW under T2, decreased by 6.09–15.13% under treatments receiving nano-N (Figure 3). GS activity varies between 10.57 and 15.52 μmol γ-GMH formed/min/g FW while GOGAT activity ranged between 2.62 and 6.02 μmol NADH oxidized/min/g FW under different N scenarios (Figure 3). The lowest GDH activity was observed under T1 (82.54 μmol NADH oxidized/min/g FW) while the highest was under T2 (129.66 μmol NADH oxidized/min/g FW). Similar results were also noticed for wheat crops (Figure 3). It is also interesting to note that rice crops had higher NR and NiR activities, while the activities of GS, GOGAT, and GDH were higher in wheat.

### 2.5. Nitrogen Availability in Soil

**Figure 4:**
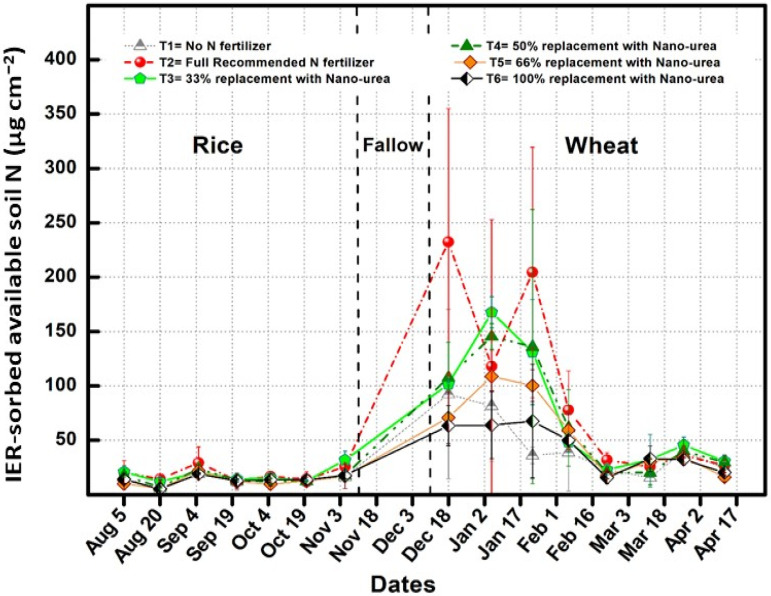
Effect of nitrogen substitution through nano-nitrogen on available soil nitrogen in rice and wheat crops.

The soil N is the most essential factor in determining the effectiveness of N in agricultural field circumstances. The ion exchange resin (IER) membranes strips were used to measure available N in the soil at fortnightly intervals and noted that during the rice crop available N was 10–30 μg cm^−2^ (Figure 4). After the harvest of rice crop, the membrane strips were removed and again inserted into the soil after 20 days of wheat sowing and higher peaks of available N were noticed during wheat season. The maximum peak of available N was noted under T2, i.e., 210–230 μg cm^−2^, followed by T3 treatment which ranged up to 170 μg cm^−2^ (Figure 4). The addition of higher nano-nitrogen followed the decreasing trend for the availability of nitrogen in soil. Substitution of more than 50% urea with nano-nitrogen lead to reduce available soil N below 100 μg cm^−2^. After incorporating a complete N dose, the available soil N showed a decreasing trend in the treatments till the harvest. It was also noticed that 100% substitution did not match with the crop requirement and leads to poor growth and yield.

## 3. Discussion

Recently, nano-fertilizers have gained relevance in sustainable agriculture for boosting crop output, improving nutrient usage efficiency, and reducing chemical fertilizer use and cultivation expenses. The appropriate choice of fertilizer, time of application, and cultivars used are crucial variables in crop management and economic output [7,8]. The present study mainly focuses on minimizing losses, as a large portion of inorganic fertilizers added to the soil are lost and become unavailable to plants. Hence, in the present study, we compare the effects of urea replacement through nano-nitrogen, and the results revealed the beneficial effects of 33% replacement of urea with nano-nitrogen which led to significant improvements in growth, plant physiology, and yield. Nano-fertilizers have a faster and higher absorption level by plants due to their small size, quick diffusion rates, and more gradual release of nutrients than their traditional counterparts, which results in superior plant development, growth, and plant biomass [9,10].

Among agronomic approaches, improving crop output with greater nutrient usage efficiency and system sustainability is based on optimizing balanced plant nutrition. Recognizing cultivar variations and key developmental phases of crops that require fertilization is crucial for effective nitrogen management. Nitrogen is a critical nutrient and a key determinant of yield, notably due to its function in photosynthesis and other biological activities such as absorption of water and minerals, vacuole storage, and xylem movement. Therefore, strategic N management might improve physical characteristics of plants by increasing metabolic efficiency. Since nano-fertilizers provide higher absorption areas and greater diffusion rates, they result in increased size and efficiency of the source, leading to higher yield attributes and grain yield [11]. The present results also showed that 33% replacement of urea through nano-N resulted in statistically on par/higher yield and yield attributes than RDN. This might be due to the large surface area and particle size of nano-urea, proving an adequate supply of nitrogen at the appropriate time which leads to higher yield [12,13]. However, replacing a higher amount of urea with nano-N led to a substantial decrease in yield attributes, indicating that the minimum amount of nitrogen required by plants to sustain productivity is not provided by nano-urea. Improvement in yield characteristics with strategic application of nano-urea might be due to effective nutrients intake (higher absorption of nutrients and their deep penetration into leaves) and metabolic output via the application of nano fertilizer, giving plants enough time to optimize nutritional efficiency and growth [14,15]. In addition to its role in activating enzymes, nano-urea also increased dry matter accumulation and growth rates, as reflected in the increase in the number of tillers, number of grains/filled grains, and grain weight.

Nitrogen accelerates leaf growth by producing proteins necessary for cell development and division, as well as being related to chlorophyll and photosynthesis. Nano-fertilizers’ controlled nutrient release provides crops with the exact amounts of nutrients, reducing stomatal resistance and increasing stomatal conductivity, thus facilitating photosynthesis and nutrient uptake, resulting in increased yield [16,17]. Additionally, sustained nutrient supply and a higher absorption capacity of nano-urea stimulated several chemical reactions via enhanced enzyme activities and reduced the influence of free radicals, leading to increased plant growth and yield [18]. The synergistic effect of nano-fertilizers along with chemical fertilizer leads to greater photosynthates accumulation and translocation to the plant’s economic parts, resulting in a high yield that was mainly credited to increased source and sink strength [19]. Nano-fertilizers enable controlled dispersion and progressive release, reducing the use of inorganic fertilizers and lessening their negative environmental impact while increasing the effectiveness of nutrient use [20].

Nitrogen metabolism controls several cellular functions in plants and significantly contributes to growth and development. N metabolism involves key enzymes like nitrate reductase (NR) and nitrite reductase (NiR), which catalyze the reduction of NO_3_^−^ to NO_2_^−^ and then NO_2_^−^ to NH_4_^+^. Further, the enzymes glutamine synthetase (GS), glutamate dehydrogenase (GDH), and glutamate synthase (GOGAT) aid in the assimilation of NH^4+^ to various amino acids and amides as well as the deamination of glutamate to produce energy and return carbon skeletons from amino acids to the reactions of carbon metabolism [21,22,23]. The present results revealed significant changes in the activities of N assimilatory enzymes and found higher activities under 100% RDN through urea than treatments receiving nano-N after 7 days of complete fertilization. This might be due to the easy and early release of nitrogen in the form of NH_4_^+^ and NO_3_^−^ through urea (2–15 days) and slow release through nano-N, influencing physiological reactions due to differing absorption and assimilation capacities [24,25]. In addition, a more rapid availability of nitrogen through urea than nano-nitrogen might increase the activity of key enzymes in nitrogen metabolism, promoting higher nitrogen accumulation in aboveground parts and impacting dry matter production [26,27]. It was also noted from literature that for the first few days of incubation, the release rate of traditional fertilizer is greater, but after that, it rapidly declines and it takes between 15 and 30 days before complete depletion is visible with conventional fertilizer. But in the case of nano fertilizer, the rate of release was greater even on the last day of incubation, despite the trend being identical to that of traditional fertilizer [28,29,30].

## 4. Materials and Methods

### 4.1. Experimental Setup and Treatment Details

A randomized block design field experiment in three replications was conducted on rice (PB 1121) during Kharif 2021 and 2022 and wheat (KRL 210) crop during Rabi 2020–2021 and 2021–2022 to evaluate the efficiency of nitrogen applied via conventional source (Urea) and dose substitution through nano-nitrogen (liquid form), provided by the Nano Biotechnology Research Center, IFFCO, Gandhinagar, India. The initial status of NPK in soil was 145–176 kg/ha N, 20.5–22.7 kg/ha P, 245–270 kg/ha K, and 0.55–0.71% OC.

Considering that one bottle of 500 mL of IFFCO Nano-N (40,000 ppm) is equivalent to 1 bag of urea (20.7 kg N), the following treatments were imposed in plots of 10.75 m × 6.0 m size. The applied N was divided into three equal splits, i.e., first dose was applied basally, second dose at 25 days after sowing (DAS), and the third dose at 45 DAS for all the treatments (Table 4). The recommended dose of nitrogen (RDN) for PB 1121 is 90 kg N/ha and KRL 210 is 150 kg N/ha. Phosphorus (SSP) and potassium (MOP) were applied basally in all the treatments. Rice seedlings (30 to 35 days old) were transplanted during the first fortnight of July and the crop was harvested during the last week of October, while the wheat crop was sown during the first fortnight of November and harvested during first fortnight of April. Other agronomic management practices, i.e., irrigation, weeding, and insect pests were uniformly followed as per recommendation for these two crops in the area.

### 4.2. Morphological and Yield Traits

Among morphological traits, plant height was recorded in five randomly selected plants per plot with a 1 m measuring scale. Non-destructive measurements of CCI (Chlorophyll Content Index) and NBI (Nitrogen Balance Index) readings were taken using Dualex Scientific+ (Force-A, Orsay, Paris, France) in randomly tagged flag leaves of rice and wheat. Yield attributes, i.e., number of tillers/mrl (meter row length), effective number of tillers/mrl recorded at physiological maturity in both rice and wheat. Panicle/spike length (cm), panicle weight (g), number of grains/spike (g), filled grain/spike (g), grain weight/spike (g), and ear-filling ratio were taken at harvest from 10 randomly selected spikes. Whereas, 1000-grain weight (g), grain yield (Kg/ha), and biological yield (Kg/ha) were recorded at harvest in both the tested crops.

### 4.3. Physiological Traits

Destructive sampling was also conducted for the measurement of chlorophyll content in the fresh leaves using the acetone method [31] and content was expressed in mg/g [32]. Relative water content (RWC) is a useful indicator of water balance which indicates the water content in percent at a given time in relation to the water content at full turgor and measured in % [33].

Total Chlorophyll Content (mg/g) = (7.49 × Abs665 + 20.34 × Abs648) × [V/(1000 × W)]

where V—volume made; W—weight of tissue.

Relative water content (%) = [(FW − DW)/(TW − DW)] × 100

where FW—Fresh weight; DW—Dry weight; TW—Turgid weight.

To monitor and relate exact changes in shape to physiological function, leaf area/plant (cm^2^) was examined in tagged leaves using a Portable Laser leaf area meter (Model CI-202, CID Bio Science, Inc. Camas, WA). Gas exchange traits, especially photosynthetic rate (Pn), transpiration rate (E), stomatal conductance (gS), and instantaneous water use efficiency (Pn/E), were measured using the Portable Photosynthesis System (LI-6800, LICOR Inc., Lincoln, NE, USA). Cuvette conditions were kept at an ambient CO_2_ concentration of 400 ppm, relative humidity > 60%, a photosynthetic photon flux density (PPFD) of 1000 μmol m^−2^ s^−1^, and leaf temperature of 25 °C [34].

### 4.4. Nitrogen-Metabolizing Enzymes

After 7 days of complete fertilizer application, fresh flag leaves samples were collected for analyzing the activities of NR (nitrate reductase) using the modified method [35]. NiR (nitrite reductase) enzyme was assayed in vivo using the method [36]. GS (glutamine synthase), GOGAT (glutamine synthase/glutamine oxoglutarate aminotransferase), and GDH (glutamine dehydrogenase) were standardized as per protocol [37].

### 4.5. Soil Available N

Ion exchange resin (IER) membranes were used to measure the soil’s available nitrogen throughout the rice–wheat growing season. The large sheets of membrane strips (anion and cation individually) were commercially available (General Electricals, Watertown, MA, USA), which were used by cutting it into small pieces of 2.5 cm by 10 cm. The strips were charged by immersing and shaking them for 1.2 h in 0.5 mol/L HCl, and then for 5 h in 0.5 mol/L NaHCO_3_. These strips were then washed in deionized water. The resin strips were inserted into a vertical slit in the treated soil and the slit was tightly closed to ensure that the strips were in contact with the soil. Both cation and anion strips were positioned 5 cm apart, left in the soil for 15 days at a time, and then removed and replaced right away with fresh ones. The strips were washed with deionized water to get rid of any remaining soil after being taken out of the soil. Cation and anion strips were kept and transferred in a vial combined for treatment to extract NH_4_^+^ and NO_3_^−^ in the lab. The strips-containing vials received 70 mL of 2 mol L^−1^ KCl for extraction. Following one hour of agitation, the resulting solution was poured into a scintillation vial through decantation. Kjeltec 2200 (Foss, Hillerod, Denmark) was used to analyze the extracts for NH_4_^+^-N and NO_3_^−^-N.

### 4.6. Statistical Analysis

Prior to data analysis, the Shapiro–Wilk (W) test was used to determine whether the observed values for each variable had a normal distribution. Using the SAS General Linear Model, analysis of variance (ANOVA) was carried out to observe morpho-physiological and yield attributes (Version 9.3, SAS Institute Inc., Cary, NC, USA). The Least Significant Difference (LSD) test was used to compare treatments at the 5% level of significance with a *p*-value of 0.05 for mean comparisons.

## 5. Conclusions

Results depicted the superiority of splitting 2nd dose of nitrogen through nano-urea (33% replacement) in both rice and wheat crops for morphological, physiological, and yield attributes over 100% N through conventional fertilizers, which was also supported by the activities of N-metabolizing enzymes. Further, it was observed that 100% replacement of urea applied through nano-N was not found satisfactory (reduction in morpho-physiological traits) in any case, either by soil application followed by foliar spray or seed priming. Nano-Nitrogen is the recent formulation of IFFCO and a partial substitution of urea dose (33%; 2nd split at 40–45 DAS) with nano-nitrogen could be considered one of the promising alternatives for Indian intensive agriculture systems (rice–wheat cropping system).

## Figures and Tables

**Table 1:** Effect of nitrogen substitution through nano-nitrogen on morphological traits of rice and wheat.

Treatments/ Traits	Rice (PB 1121)			Wheat (KRL 210)		
	Plant Height (cm)	CI	NBI	Plant Height (cm)	CI	NBI
Year (Y)						
2021	91.38 ^B^	28.89 ^B^	28.33 ^B^	92.73 ^B^	24.46 ^B^	30.57 ^B^
2022	101.22 ^A^	34.39 ^A^	33.68 ^A^	99.43 ^A^	29.53 ^A^	37.24 ^A^
CD @ 5% (Y)	1.94	0.48	0.49	1.72	0.41	0.64
Treatments (T)						
T1	78.5 ^D^	25.01 ^D^	22.3 ^F^	79.33 ^C^	23.90 ^E^	28.87 ^E^
T2	101.88 ^A^	35.99 ^A^	34.6 ^B^	102.17 ^A^	28.02 ^B^	36.98 ^B^
T3	102.5 ^A^	33.96 ^B^	36.7 ^A^	103.17 ^A^	30.25 ^A^	39.28 ^A^
T4	101.12 ^A^	33.7 ^B^	33.7 ^C^	102.0 ^A^	27.53 ^C^	34.77 ^C^
T5	100.62 ^B^	33.34 ^B^	30.8 ^D^	101.83 ^A^	27.17 ^C^	34.10 ^C^
T6	91.78 ^C^	27.83 ^C^	28.7 ^E^	89.55 ^B^	26.98 ^D^	30.98 ^D^
CD @ 5% (T)	3.36	0.83	0.85	2.98	0.71	1.11
CD @ 5% (Y × T)	4.75	1.18	NS	NS	NS	1.57

Means followed by at least one letter common are not statistically significant (*p* < 0.05).

**Table 2:** Effect of nitrogen substitution through nano-nitrogen on yield-attributing traits of rice and wheat crop.

Treatment/ Traits	Rice (PB 1121)								Wheat (KRL 210)							
	No. of Tillers	No. of Effective Tillers	Panicle Length	Panicle Weight	No. of Grains/ Panicle	No. of Filled Grains	Grain Weight/ Panicle	Ear Filling Ratio	No. of Tillers	No. of Effective Tillers	Spike Length	Spike Weight	Spikelets/Spike	No. of Grains/ Spike	Grain Weight/ Spike	Ear Filling Ratio
Year (Y)																
2021	50.80 ^B^	40.09 ^B^	8.32 ^B^	1.94 ^B^	88.88 ^B^	75.25 ^B^	1.53 ^B^	0.59 ^B^	45.89 ^B^	39.85 ^B^	8.45 ^B^	2.23 ^B^	15.63 ^B^	46.87 ^B^	1.70 ^B^	0.59 ^B^
2022	60.07 ^A^	46.15 ^A^	10.32 ^A^	2.74 ^A^	98.87 ^A^	85.25 ^A^	1.73 ^A^	0.79 ^A^	51.89 ^A^	47.85 ^A^	10.45 ^A^	3.03 ^A^	17.96 ^A^	51.37 ^A^	1.98 ^A^	0.77 ^A^
CD @ 5% (Y)	0.96	0.74	0.18	0.04	1.79	1.21	0.02	0.011	0.91	0.58	0.22	0.04	0.32	0.87	0.03	0.01
Treatments (T)																
T1	35.75 ^F^	31.50 ^E^	6.98 ^G^	1.77 ^F^	79.50 ^C^	53.50 ^F^	0.98 ^E^	0.55 ^C^	37.33 ^D^	31.17 ^F^	7.63 ^E^	2.02 ^E^	13.73 ^F^	37.67 ^E^	0.91 ^E^	0.45 ^D^
T2	60.50 ^A^	49.50 ^A^	10.45 ^AB^	2.61 ^AB^	103.00 ^AB^	92.00 ^A^	2.01 ^A^	0.77 ^A^	54.92 ^AB^	49.83 ^AB^	10.49 ^A^	2.88 ^A^	17.90 ^AB^	53.80 ^AB^	2.26 ^B^	0.79 ^A^
T3	59.25 ^A^	48.75 ^A^	10.85 ^A^	2.70 ^A^	104.00 ^A^	95.00 ^A^	2.05 ^A^	0.76 ^A^	55.75 ^A^	50.25 ^A^	10.38 ^AB^	2.85 ^A^	18.77 ^A^	55.23 ^A^	2.31 ^A^	0.81 ^A^
T4	52.25 ^BC^	47.50 ^A^	10.03 ^ABC^	2.53 ^BC^	94.25 ^ABC^	88.25 ^AB^	1.87 ^B^	0.74 ^AB^	53.33 ^AB^	48.75 ^ABC^	9.95 ^C^	2.81 ^B^	17.37 ^BC^	53.13 ^AB^	2.15 ^BC^	0.76 ^B^
T5	50.00 ^BCD^	43.00 ^BC^	9.53 ^BCD^	2.34 ^D^	93.50 ^ABC^	85.00 ^ABCD^	1.65 ^C^	0.71 ^B^	52.08 ^AB^	47.83 ^BC^	9.84 ^C^	2.72 ^C^	17.53 ^BC^	51.57 ^ABC^	2.05 ^C^	0.75 ^B^
T6	39.00 ^E^	39.00 ^BCD^	8.08 ^EF^	2.07 ^E^	89.00 ^ABC^	67.75 ^E^	1.21 ^D^	0.58 ^C^	39.92 ^D^	35.25 ^E^	8.43 ^D^	2.53 ^D^	15.47 ^D^	43.33 ^D^	1.37 ^D^	0.54 ^C^
CD @ 5% (T)	1.67	1.29	0.31	0.07	3.11	2.10	0.04	0.020	1.58	1.01	0.39	0.06	0.55	1.50	0.06	0.02
CD @ 5% (T × Y)	NS	1.82	NS	NS	NS	NS	NS	NS	NS	NS	NS	NS	NS	NS	0.08	NS

Means followed by at least one letter common are not statistically significant (*p* < 0.05).

**Table 3:** Effect of nitrogen substitution through nano-nitrogen on physiological traits of rice and wheat crops.

Treatment/ Traits	Rice (PB 1121)					Wheat (KRL 210)				
	Chlorophyll Content (mg/g)	Pn (μmol m^−2^ s^−1^)	gS (mol m^−2^ s^−1^)	E (mmol m^−2^ s^−1^)	WUEi (μmol/ mmol)	Chlorophyll Content (mg/g)	Pn (μmol m^−2^ s^−1^)	gS (mol m^−2^ s^−1^)	E (mmol m^−2^ s^−1^)	WUEi (μmol/ mmol)
Year (Y)										
2021	1.83 ^B^	12.52 ^B^	0.21 ^B^	3.85 ^B^	3.37 ^B^	1.89 ^B^	11.54 ^B^	0.32 ^B^	3.61 ^B^	3.33 ^B^
2022	2.02 ^A^	18.72 ^A^	0.38 ^A^	4.07 ^A^	4.63 ^A^	2.09 ^A^	16.03 ^A^	0.54 ^A^	4.01 ^A^	4.06 ^A^
CD @ 5% (Y)	0.031	0.265	0.009	0.078	0.104	0.034	0.248	0.009	0.069	0.11
Treatments (T)										
T1	1.56 ^E^	9.61 ^E^	0.19 ^E^	2.31 ^E^	4.16	1.71 ^C^	10.27 ^E^	0.18 ^F^	2.22 ^E^	4.63
T2	2.12 ^AB^	17.75 ^A^	0.38 ^A^	4.98 ^A^	3.56	2.14 ^A^	15.39 ^B^	0.55 ^A^	4.41 ^AB^	3.49
T3	2.15 ^A^	17.87 ^A^	0.38 ^A^	4.74 ^A^	3.77	2.17 ^A^	16.20 ^A^	0.53 ^AB^	4.68 ^A^	3.46
T4	2.02 ^B^	16.37 ^B^	0.31 ^B^	4.26 ^B^	3.84	2.07 ^AB^	14.50 ^B^	0.48 ^BC^	4.21 ^B^	3.44
T5	1.95 ^C^	16.03 ^C^	0.27 ^C^	4.04 ^C^	3.97	2.02 ^B^	13.81 ^C^	0.43 ^CD^	4.06 ^C^	3.40
T6	1.74 ^D^	15.50 ^D^	0.23 ^D^	3.46 ^D^	4.48	1.80 ^C^	13.52 ^D^	0.34 ^E^	3.21 ^D^	4.21
CD @ 5% (T)	0.054	0.459	0.005	0.136	0.179	0.059	0.429	0.015	0.119	0.20
CD @ 5% (T × Y)	0.076	0.649	0.012	NS	0.245	0.084	0.607	0.021	0.169	0.28

Means followed by at least one letter common are not statistically significant (*p* < 0.05).

**Table 4:** Treatments details.

S. No.	Treatment	Basal Dose (1/3rd N)	At 25 Days after Sowing (DAS) (1/3rd N)	At 45 Days after Sowing (DAS) (1/3rd N)
1.	Without Nitrogen—(T1)	-	-	-
2.	Recommended dose of nitrogen (RDN)—(T2)	Urea	Urea	Urea
3.	33% replacement of N − ⅓ N through urea + ⅓ N through urea + ⅓ N through nano-nitrogen (T3)	Urea	Urea	Nano-N
4.	50% replacement of N − {⅓ N through urea + (½ N through Urea + ½ N through nano-nitrogen) + ⅓ N through nano-nitrogen} (T4)	Urea	50:50 (Urea + Nano-N)	Nano-N
5.	66% replacement of N − ⅓ N through urea + ⅓ N through nano-nitrogen + ⅓ N through nano-nitrogen (T5)	Urea	Nano-N	Nano-N
6.	100% replacement of N − ⅓ N through nano-nitrogen + ⅓ N through urea + ⅓ N through nano-nitrogen (T6)	Nano-N (Seed priming)	Nano-N	Nano-N

## Data Availability

The data will be provided on reasonable request from the corresponding authors.
